# Membrane Emulsification—A Novel Solution for Treatment and Reuse of Produced Water from Oil Field

**DOI:** 10.3390/membranes12100971

**Published:** 2022-10-02

**Authors:** Aamer Ali, Usman Taqui Syed, Thomas Skovfoged Bak, Cejna Anna Quist-Jensen

**Affiliations:** 1 Center for Membrane Technology, Department of Chemistry and Bioscience, Aalborg University, Fredrik Bajers Vej 7H, 9220 Aalborg, Denmark; 2 LAQV/Requimte, Department of Chemistry, NOVA School of Science and Technology, FCT NOVA, Universidade NOVA de Lisboa, 2829-516 Caparica, Portugal

**Keywords:** oil-in-water emulsions, membrane emulsification, hollow fiber, enhanced oil recovery

## Abstract

Produced water (PW) is, by volume, the largest waste product of the oil- and gas-exploration industry and contains pollutants such as hydrocarbons and heavy metals. To meet the stringent environmental regulations, PW must be treated before discharging into the environment. The current study proposes a novel treatment method where PW is used to prepare oil-in-water emulsion with potential applications within the oil-exploration industry. The emulsions are prepared by applying hollow fiber membrane emulsification (ME) on PW, which inherently contains oil, as to-be-dispersed phase. The results demonstrate that the average droplet size of the emulsions is a function of pressure applied on to-be-dispersed phase and could be customized from 0.24 to 0.65 µm by varying the pressure from 0.25 to 1 bar, respectively. Stability of the emulsions was verified under high pressure and a temperature and storage period of more than 24 h. The calculations showed that an ME unit with <100 kg weight and <1 m^3^ volume is appropriate to transform the daily average volume of PW from the Danish part of the North Sea into the emulsions. The study provides a novel route, which also complies well with the requirements (low-weight and small spatial footprints) of the offshore oil rigs, to treat and reuse PW within the oil production process and, therefore, eliminates its environmental footprint.

## 1. Introduction

Despite the huge efforts to switch to green energy, oil is expected to play a crucial role in meeting global energy demands, at least in the near future. The oil-exploration process, at the same time, generates huge volume of produced water (PW) which is separated from the oil using a series of separation units [1,2]. PW separated from the oil contains pollutants including heavy metals, residual oil and greases and several other organic and inorganic compounds [2]. The treatment of produced water before discharge, therefore, is important from an environmental perspective. Traditionally, various physical, chemical and biological methods have been tried to treat produced water to meet discharge requirements [3,4,5,6,7]. However, there is great interest in developing methods that minimize the need to discharge PW, and thereby eliminate its environmental impact [8].

Crude-oil-in-water emulsions find numerous applications in the oil and gas industry such as drilling, enhanced oil recovery, hydraulic fracturing and removal of oil from produced water. Applications, such as enhanced oil recovery and removal of oil from PW through demulsification, require specifically tailored emulsion properties such as droplet size and size distribution and chemistry [9,10]. In particular, for enhanced oil recovery, the emulsion droplets within oil-containing porous rocks are captured according to the filtration mechanism where droplet-to-pore-diameter ratio becomes the governing parameter for retention [11,12]. The dispersed phase in oil-in-water emulsions effectively blocks the channeling through high-permeable zones and forces the displacing fluid to flow through the un-swept regions, leading to EOR by improving reservoir sweep and mobilizing trapped residual oil [13,14,15]. The other properties of the emulsions, such as viscosity, type and concentration of surfactant and the resultant oil–water interfacial tensions, are also important in this regard [16,17].

Several methods, such as high-shear mixers, high-pressure homogenizers and sonicators are reported in the literature for the formation of oil-in-water or water-in-oil emulsions. However, these methods are highly energy intensive and generally lack the ability to control the droplet size and distribution [18,19]. As much as 95% of the energy input is lost as heat, which can lead to the degradation of heat-sensitive ingredients [20,21]. The efficiencies are even lower for the techniques applied for the production of sub-micrometer emulsions that also generate high temperatures, which contribute to further incompatibility with pharmaceutical, cosmetic, food components and petroleum products [22,23]. Therefore, the design of new and improved technologies might help to overcome the obstacles observed in the preparation of tailored emulsions.

Membrane emulsification (ME) is a technique that offers an alternative to traditional methods for the production of emulsions in a highly efficient manner. The concept of ME was developed in the late 1980s by Nakashima et al., who initially produced oil-in-water and water-in-oil emulsions [24,25]. Reduction in energy utilization, control over droplet size and size distribution and potentially easy scaling up/down are the main distinguishing features of the ME process [26]. Recent studies by Syed et al. demonstrated the efficiency of the process in producing smaller and stable emulsions along with enhanced energy savings of the membrane emulsification technique over the conventional ultrasound emulsification technique. Comparative studies evaluated the energy density to emulsions, whereby membrane-based process ensures a 99.5% reduction in energy expenditure to produce the same surface area by sonicators [18,27].

The formation of emulsion during ME can primarily be achieved in two ways: (i) by pressing a coarse premixed phase through the membrane to reduce the droplet size of the dispersed phase and (ii) by pushing a to-be-dispersed phase through the membrane that forms droplets on pore-mouth on the other side of the membrane and the droplets are subsequently detached from the membrane surface by cross flow of the continuous phase [28,29]. These two techniques are called dead-end or premix ME and direct or cross-flow ME, respectively. In direct ME, fine droplets are formed in-situ at the interface of the membrane and continuous phase. However, this technique suffers from the low flux of dispersed phase through the membrane which must be maintained to avoid the transition from size-stable to a “continuous outflow” zone and to avoid the steric hindrance among the droplets formed at the adjacent pores [30,31,32]. In addition, shear stress should be applied on the permeate side of the membrane to detach the droplets from the surface, which results in the possibility of additional energy requirements. Premix ME, on the other hand, offers some unique advantages: the optimum trans-membrane flux can be up to one to two orders of magnitudes higher than that for the direct ME, which directly translates into small and light weight setups, smaller mean droplet sizes compared to direct ME under the same operating conditions, simpler experimental set-ups and an easy-to-control process [33]. In both the configurations, droplet size and size distribution can be controlled by tuning-membrane and process parameters such as the size, size distribution and uniformity of the membrane pores, membrane material (hydrophobic or hydrophilic), applied pressure and cross-flow velocity of the continuous phase, to name a few.

Due to the aforementioned advantages, ME has been investigated for several applications. For instance, ME has been investigated to tune the particle size distribution of drug-loaded polymer microspheres to control the rate and duration of drug release [34]. In the food industry, ME is used as a mild and energy-efficient process to prepare different oil-in-water or water-in-oil emulsions [35]. ME has also been studied for the preparation of emulsions with applications as an ingredient used in cosmetic products [28]. However, the studies on ME for the fabrication of crude oil/water emulsion, which have numerous potential applications within the oil-exploration industry, are still scarce. The focus of the membrane-based studies carried out in this regard is the removal of oil droplets from wastewater streams and not the formation of tailored emulsions [36,37,38,39]. Therefore, a better understanding of the correlation of emulsion properties with the membrane and process parameters for ME application needs to be explored. The existing literature also reveals that ME is mainly carried out using Shirasu Porous Glass (SPG) [40,41], metallic [42,43] and ceramic membranes [44,45]. These membranes are expensive and have low productivity and, therefore, are not suitable for the commercial-scale production of the crude oil-in-water emulsions for offshore installations, which must fulfil the criteria of being economic, lightweight and compact (small physical footprints). Hollow-fiber polymeric membranes, which are a well-acknowledged solution for commercial applications due to their high productivity and easy assembling into the module, have been merely studied for ME.

In the current study, we present a novel alternative to the traditional PW treatment that exploits oil present in PW to generate oil-in-water emulsions with potential applications within the oil-exploration industry (enhanced oil recovery, demulsification and drilling). Pure water as well as seawater were used as the continuous phase, whereas PW was used as to-be-dispersed phase. Commercial hollow-fiber polymeric membranes were used to prepare the emulsions through ME. The emulsions were characterized in terms of droplet size and size distribution, and the stability in terms of temperature, pressure and storage period was evaluated. The effects of the salinity of the continuous phase and applied pressure on the droplet size was also investigated.

## 2. Materials and Methods

### 2.1. Membranes

The emulsion experiments were carried out using polypropylene (PP) hollow-fiber membranes (Membrana Accurel^®^ PP S6/2) assembled into a transparent plastic module. A detailed description of the applied membrane fibers and modules can be found in Table 1. 

### 2.2. Finding the Suitable Membrane Pretreatment and Operational Mode of ME

As-received PP membrane demonstrates hydrophobic character, however, the preparation of oil-in-water emulsions requires that membrane should exhibit hydrophilic character. Therefore, a hydrophilic pretreatment was required to make it suitable for preparing the emulsions, which was achieved according to two schemes: (i) in the first pretreatment scheme, hydrophilic pretreatment suggested by the manufacturer was applied, i.e., 30 % isopropanol solution was circulated into the membrane module for 30 minutes followed by the cleaning of membrane with distilled water; and (ii) in the second pretreatment scheme, the membranes initially pretreated with 30 % isopropanol solution were further activated by pushing a 2 % crude (without any surfactant) oil-in-water mixture, homogenized by rigorous mixing with magnetic stirrer, through the membrane pores continuously for two hours followed by cleaning the membrane according to the procedure described in Section 2.7. 

### 2.3. Membrane Emulsification

**Figure 1:**
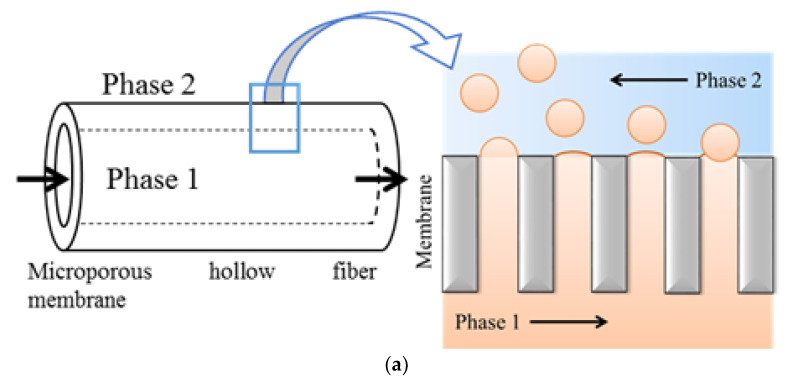

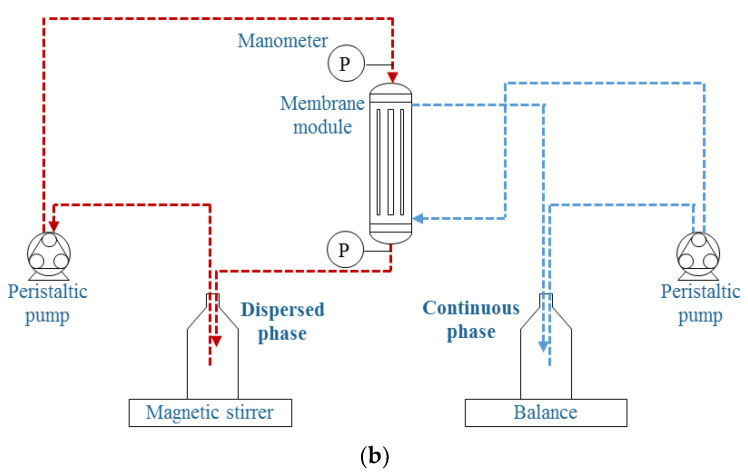
(**a**) Schematic representation of membrane emulsification process; and (**b**) membrane emulsification setup.

The membrane emulsification set up used in this study is shown in Figure 1. In the main experiments (excluding those reported in Section 3.1), the continuous and dispersed phases were introduced on the shell side and lumen side of the fibers, respectively. The flow rate of the continuous phase was kept constant at 45 L/h, corresponding to a velocity of 0.26 m/s. The dispersed phase used in the main experiments consisted of 1% crude oil from the North Sea (provided by the Danish Hydrocarbon Research and Technology Centre) mixed with 1% dispersant and 98% distilled water. From practical point of view, PW with this oil concentration can be obtained from an appropriate point along the oil–water separation train. The dispersant was prepared by mixing 40% (*w*/*w*) ethanol with 60% (*w*/*w*) surfactant (Tween 80 and Lecithin in ratio of 3:2) [46]. All the reagents used were commercial grade and were purchased from Sigma Aldrich. The initial volume of each phase was 500 mL. To investigate the effect of pressure on droplet size distribution of the dispersed phase, the pressure on dispersed phase was varied to 0.25, 0.5 and 1 bar in a series of experiments where distilled water was used as the continuous phase. The maximum value of applied pressure was limited to 1 bar to mimic the real-life scenario where the available pressure at the end of oil–water separation line is small (≤1 bar). To study the effect of salt content on the emulsion properties, the continuous phase was replaced with artificial seawater (3.5 g/L NaCl) in one of the experiments. For each experiment, the flux and droplet size were recorded. The effect of ME configuration, including direct ME and premix ME, on the emulsion formation process was scrutinized during initial experiments. 

### 2.4. Droplet Size Measurement

The size distribution of the emulsion droplets was measured using dynamic light scattering (DLS) (Malvern ZETASIZER) technique. The DLS measurements were carried out using oil as the material and water as the dispersant. The temperature was set to 22 °C and the equilibration time was set to 30 seconds. The annotator was selected automatically by the instrument. During the experiments, samples were taken for analysis every 40 minutes. The formation of the emulsions was also monitored by using an optical microscope (ZEISS, Axioskop) with camera (Infinity X). The size of the oil droplets in water phase was measured by the software ImageJ. 

### 2.5. Stability of Emulsions

Stability of the emulsions under various rotation speeds and temperatures was analyzed by a LUMiSizer—Dispersion Analyzer (LUM GmbH). The instrumental parameters used were: volume of emulsion: 1.8 ml; from 200–4000 rpm; total run time: 600 s; time interval: 10 s; number of profiles: 10; temperature: 25 °C and 50 °C. 

### 2.6. Stability of the Membrane Fibers

It was observed that the membrane fibers became curly in shape (see Section 3.5) during the emulsion experiments. In order to probe the reason for curling, 5 cm long pieces of the virgin membrane fiber were immersed in 4 different solutions (20 wt % crude oil, 1 wt % crude oil, 0.5 wt % crude oil and deionized (DI) water). The four samples were placed in a rotational mixer and the length of the fibers were measured after 0.5 h, 1 h, 4 h and 144 h (6 days).

### 2.7. Cleaning Procedure

After each experimental run, the membrane module was cleaned by a three-step process. Initially, the setup was flushed with deionized (DI) water to remove the bulk of the remaining oil-in-water mixture from the membrane surface. Following this, an alkaline solution consisting of DI water with 0.05 wt % EDTA, 0.2 wt % SDS and 0.2 wt % sodium pyrophosphate was used for 30 minutes. The pH was adjusted to 11 with a 2 M sodium hydroxide solution. The purpose of the alkaline cleaning solution was to remove any oil stuck to the membrane surface. Finally, the module was rinsed with DI water until neutral conditions were established.

## 3. Results and Discussion

### 3.1. Selection of the Appropriate Pretreatment and ME Configuration

In the first stage of the pretreatment, hydrophilic membranes were obtained by pretreating the membranes with 30% isopropanol solution according to the procedure described in Section 2.2. Direct ME experiments were carried out in continuous mode by introducing pure crude oil on the lumen side and distilled water along with the dispersant as the continuous phase on the shell side. However, a significant quantity of the crude oil came out of the membrane under very mild pressure (<0.2 bar), and adhered to the membrane and covered the entire surface making the emulsification impossible. In the next stage, the experimentation was performed using a premix ME configuration where 1% crude oil along with the dispersant, stirred continuously at 300 rpm using a magnetic stirrer, was introduced on the lumen side of the module. In this case, some small oil droplets in the continuous phase were observed initially, however, after a few minutes, the surface of the membrane was completely covered with the oil which, similar to the initial experiments, started releasing big droplets in the continuous phase. 

In the next stage, the membranes pretreated according to the second pretreatment scheme (see Section 2.2) were applied and it was observed that these membranes did not show any tendency to adsorb the oil on its surface and was suitable for further experiments. It was hypothesized that pendant groups present on the surface of PP membrane interacted with crude oil fractions through hydrophobic–hydrophobic interactions. These interactions result in the adsorption of the oil fractions on the membrane surface, which enhances its hydrophilic character. The hypothesis was also supported by the FTIR spectrum (Figure A4 in Appendix A) of the membrane before and after the pretreatment, where a clear change was seen in peaks related with the stretching of CH_3_ group (2950, 2918, and 2836). This is in accordance with the literature, where incomplete desorption of the crude oil constitutes from PP surfaces has been reported even after washing the membrane [47]. The adsorption of oil constituents on the membrane surface was also evident from the color of the hollow fiber, which remained brown even after the alkaline cleaning (see Section 3.5). The increase in hydrophilicity was confirmed from the pure-water flux, which doubled after pretreatment with oily water compared to simple pretreatment with isopropanol. The membrane pretreated according to this procedure was used in the further studies reported hereafter.

### 3.2. Total Flux

**Figure 2:**
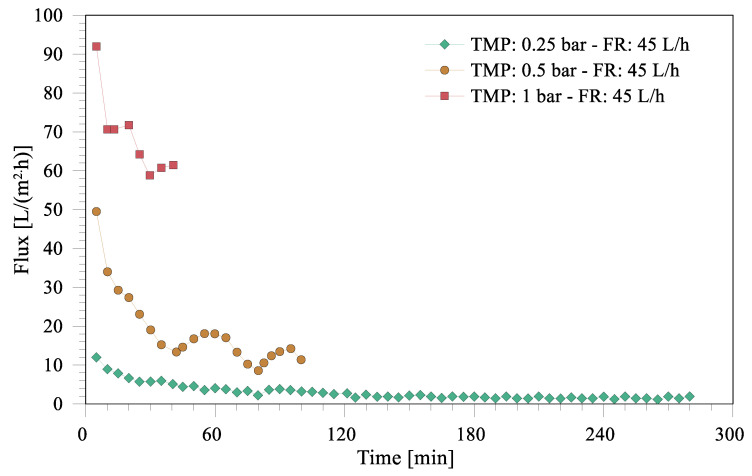
Flux over experimental time for ME experiments carried out at different pressures. FR represents flow rate.

The variation in the flux of the dispersed phase through the membrane over time for the experiments carried out at different pressures is shown in Figure 2. As expected, higher trans-membrane pressure yields higher transport through the membrane (flux), as suggested by Darcy’s law. The average flux over the experiments carried out at 1, 0.5 and 0.25 bar were 68.6 ± 10.8, 17.8 ± 9.0 and 3.1 ± 2.5 L/m^2^·h, respectively. The experiments were performed to push 50% of the initial volume of the premixed oil–water mixture through the membrane; therefore, the experimental time was different for different pressures due to the different rates of transport through the membrane. 

**Figure A1:**
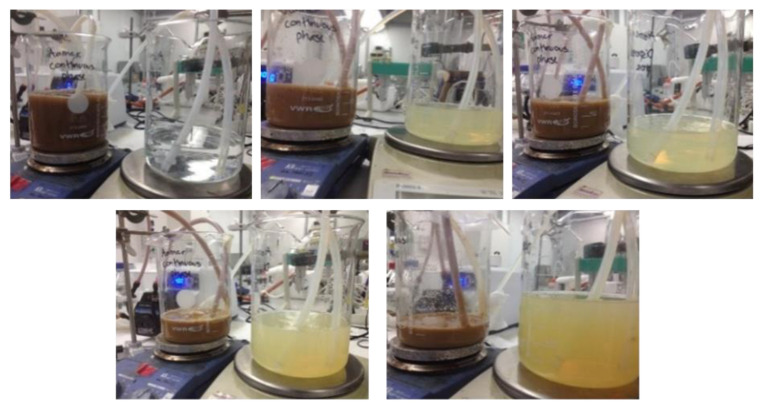
Emulsion formation as function of experimental time (from 0–3.5 h) for the experiments carried out at 0.25 bar pressure.

It is also evident from Figure 2 that the flux drops down with experimental time for all three pressure conditions. Total decrease in flux for 0.25, 0.5 and 1 bar applied pressure was 88, 76 and 35 %, respectively. The observed trend in the flux can be attributed to the possible deposition of oil droplets of various sizes at the membrane surface. It is well-acknowledged in the literature that, for a given pore size and transmembrane pressure, there exists a critical droplet size; the droplets that are bigger than the critical size are retained by the membrane and the smaller droplets permeate through the pores [48]. At low applied pressure, only small droplets pass through the membrane pores and large droplets are retained at the membrane surface, which eventually form a stable fouling layer at the surface. The highest overall decrease in flux at low pressures can be attributed to the potentially more stable fouling-layer buildup under these conditions. As the experiment proceeds, the concentration of these droplets at the membrane surface keeps on increasing, contributing to the fouling resistance that further reduces the flux. This was essentially what was observed for the flux at 0.25 bar where the flux kept on declining over experimental time. Higher transmembrane pressures (0.5 and 1 bar) more effectively push the oil from the membrane surface into the membrane pores and, therefore, the buildup of a stable fouling layer becomes difficult; thus, less decrease in flux is observed under these pressure conditions. It was also observed that the color of the continuous phase changed from transparent to blackish yellow during the experiment (see Figure A1 in Appendix A) due to an increase in the oil concentration over experimental period. 

### 3.3. Droplet Size and Size Distribution

#### 3.3.1. Effect of Applied Pressure on Droplet Size and Size Distribution

**Figure 3:**
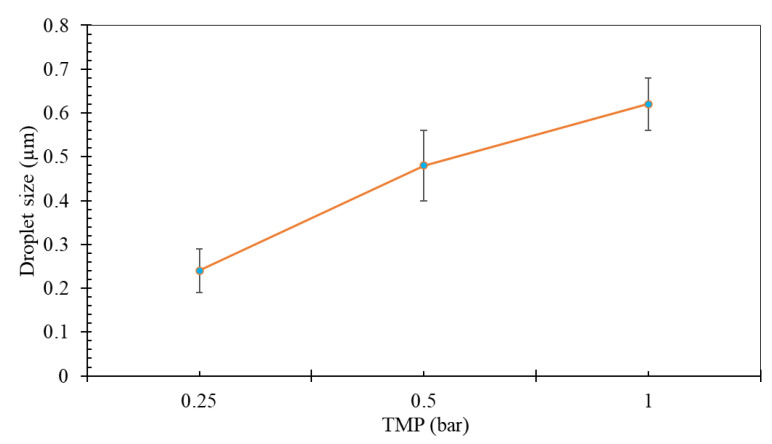
Average droplet size as function of applied pressure.

The samples were analyzed for their emulsion droplet size using DLS. The average droplet sizes for the experiments carried out under different pressures are shown in Figure 3. It is evident from the figure that the droplet size increases with the applied pressure on the premixed phase. The minimum average droplet size (0.24 µm) observed in the study is close to the average pore size of the membrane (0.2 µm) and it reaches as high as three times the membrane pore size when the pressure is increased to 1 bar. A droplet size bigger than the membrane pore size observed in all the cases indicates that coalescence of the droplets took place, which yielded the droplet sizes bigger than the membrane pore size [44]. The observed trend of the average droplet size as function of the applied pressure is consistent with the discussion provided for the flux in Section 3.1. In pores where the dispersed phase passes in the form of the droplets, under high pressures, even the bigger droplets pass through the membrane pores due to their deformation at the pore mouth. Upon exiting the pore, the emulsion droplets are deformed again to regain their spherical shape, as discussed in more detail in the literature [30]. The observed trend is also coherent with the literature [49], which suggests that the high pressure transfers more mass to the droplet before detachment from the membrane surface and, therefore, increases the droplet size [50]. Moreover, the results depicted in Figure 3 are in accordance with the literature, wherein smaller dispersed phase flux offers better control over the emulsion droplets size and dispersity [19,51,52]. Bigger droplets and more oil passing through the membrane pores also favors the coalescence of the droplets, which is possibly another factor contributing to the observed large droplet sizes under high-pressure conditions. Hence, to curtail the coalescence of the emulsion droplets, the emulsification experiments need to be performed at a lower dispersed phase flux and at higher continuous phase wall shear stress by increasing the crossflow velocity of the continuous phase [53,54]. The application of low applied pressure also corresponds to the real-life scenario where the pressure of produced water near the end of the oil–water separation train is very low (≤1 bar). 

**Figure 4:**
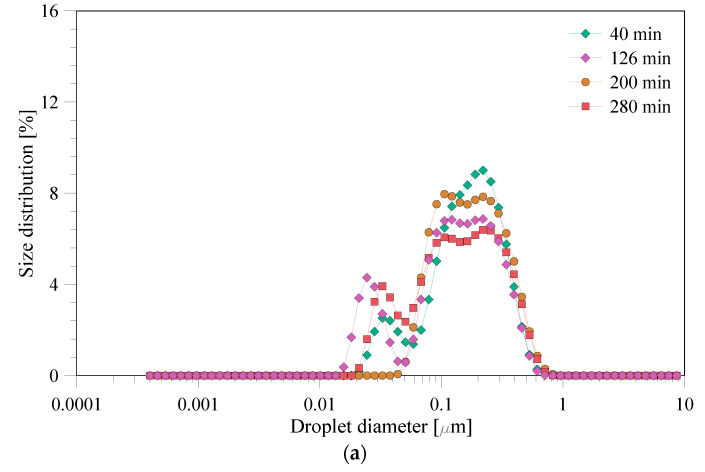

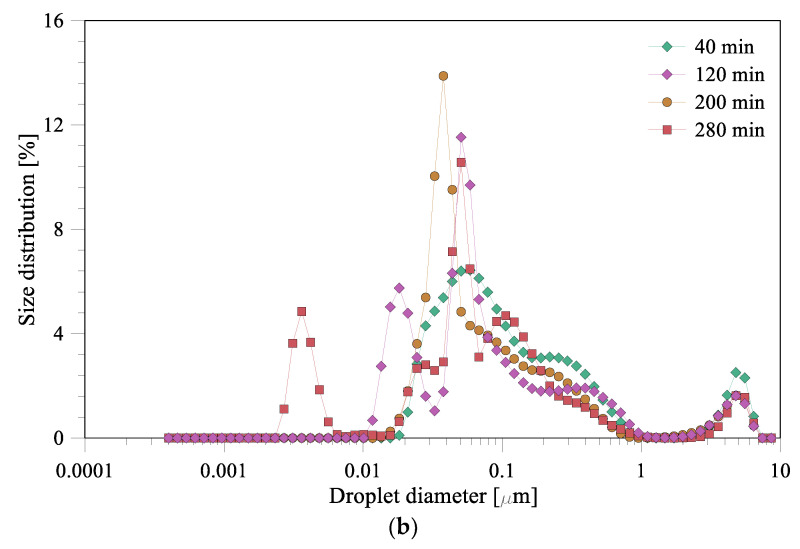
Droplet size distribution measured for applied pressures of (**a**) 0.25 and (**b**) 1 bar.

The droplet size distributions for the experiments at 0.25 and 1 bar are shown in Figure 4a,b, respectively. The samples were withdrawn at different times over the experimental period for the droplet size distribution analysis and were analyzed with DLS. As can be noticed for the experiment at 0.25 bar, there are two clear peaks for all the samples, whereas for the 1 bar experiment there are more peaks. The droplet size changed slightly over the experimental period; however, there was no clear trend. For instance, the droplet size distribution for the emulsions prepared at 0.25 bar pressure slightly retreats from its mean value over experimental period. On the other hand, for the emulsions prepared at 1 bar, the droplet size distribution becomes narrower with time.

#### 3.3.2. Effect of Composition of the Continuous Phase on Droplet Size

**Figure 5:**
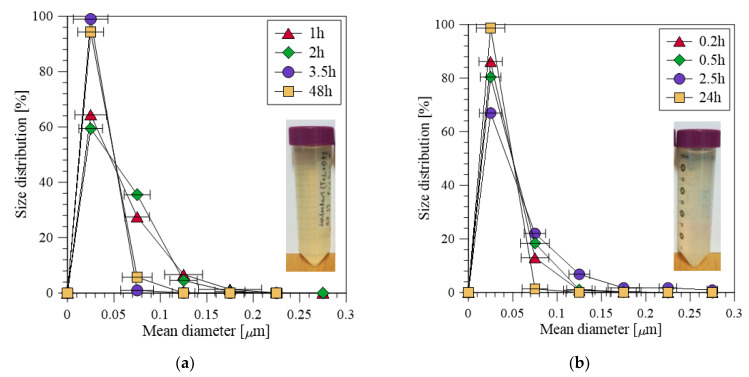
Droplet size distribution of emulsions (**a**) in distillate water and (**b**) in 3.5% NaCl solution at applied pressure of 0.25 bar.

Salinity level of oil-in-water emulsions is considered as an important quality parameter in applications such as water injection for EOR [55]. To study the effect of the composition of the continuous phase on emulsion properties, distilled water as well as 3.5 % NaCl solution was used as the continuous phase, whereas 1% crude oil along with the dispersant was applied as to-be-dispersed phase. As such, prepared emulsions were also used to further study the effect of the storage time on the droplet size distribution. The droplet size distribution of the emulsions prepared by using the two types of the continuous phases is shown in Figure 5a,b. It can be noted from the figures that the droplet size distribution does not change significantly during the first 2 h; however, a slight change is observed at 3.5 h for distillate water as the continuous phase, where the droplet size distribution becomes narrower. This can be due to further breakage of the droplets during their continuous circulation into the system over the experimental period. No significant change is observed after storing the two emulsions for 24 and 48 h. At lab scale, the emulsification process was carried out in batch mode, but in real application the emulsification will be a continuous process. It can also be noted from the figures that the emulsion in the NaCl solution seems to have a narrower size distribution compared to the emulsion in the distillate water, which suggest that the salt solution together with the surfactants stabilize the emulsions. This aspect has also been discussed in one of our previous publications, where it was inferred that the increased ionic concentration allows the surfactants to be packed more efficiently and, therefore, decreases the protective electrical double layer (measured through zeta-potential) at the hydrophilic parts of the emulsifier, which results in more stabilized oil-in-water emulsions [7]. 

**Figure A2:**
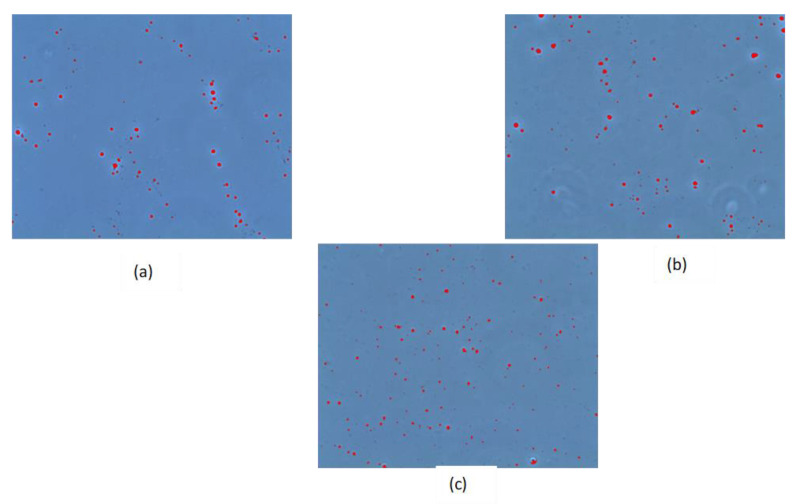
Image examples of emulsions in distillate water (**a**) sample after 1 h, (**b**) sample after 3.5 h, (**c**) sample after experiment at 48 h.

**Figure A3:**
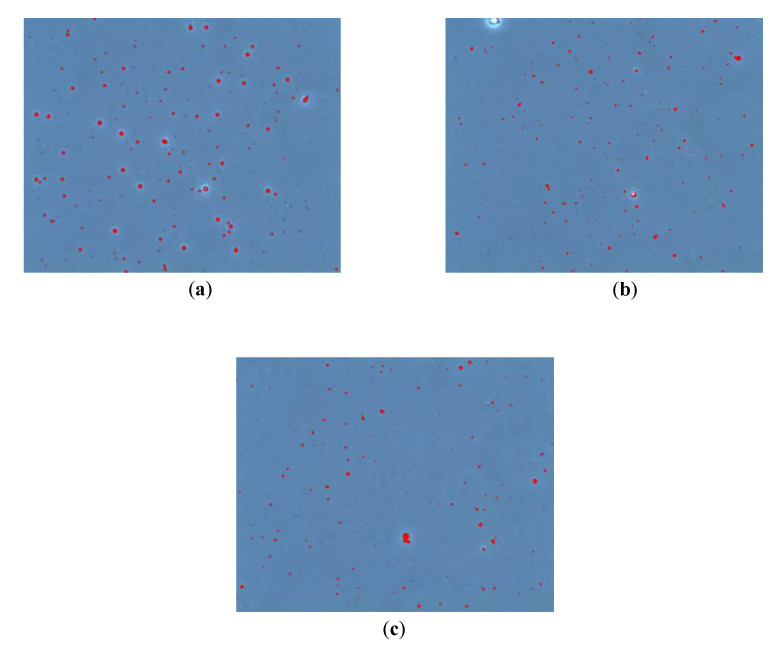
Image examples of emulsions in 3.5% NaCl solution (**a**) sample after 0.5 h, (**b**) sample after 2.5 h, (**c**) sample after experiment at 24 h.

**Figure A4:**
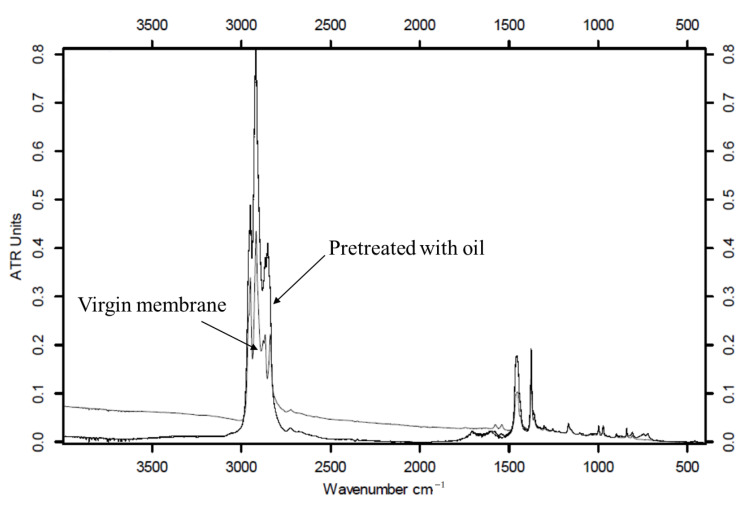
FTIR spectra of virgin and pretreated with oil membrane.

During these experiments, samples were withdrawn from the feed container and analyzed by optical microscope. The images of formed emulsions recorded through the microscope during and after the experiment are shown in Figure A2 and Figure A3 in the Appendix A for distilled water and 3.5 % NaCl solution, respectively, used as continuous phases. In these images, the oil droplets and their approximate distribution can be seen. 

### 3.4. Stability under High Rotation Speed and Temperature

**Figure 6:**
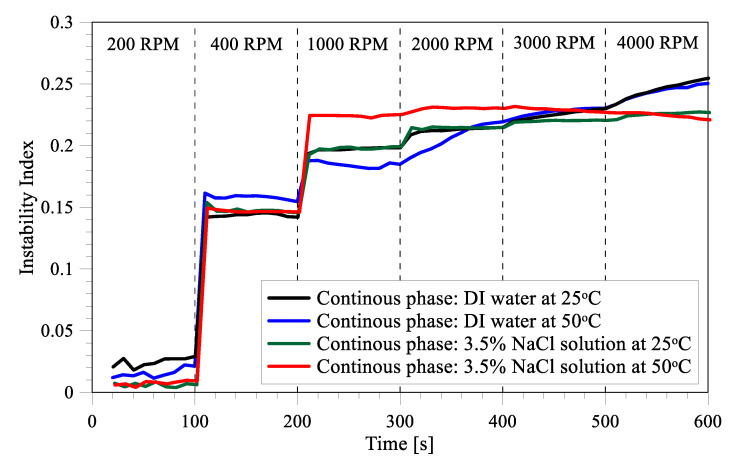
Instability index of the prepared emulsions at various rotational frequencies and temperature of 25 °C and 50 °C.

The long-term stability of the prepared emulsions was analyzed by a LUMisizer that estimates the instability index (creaming effect). The instability index is used to characterize the emulsion stability. It is measured as a ratio of the clarification at any given time divided by the maximum clarification. The clarification is quantified in terms of increase in transmission resulting from phase separation due to sedimentation or creaming/floatation. Instability index is a dimensionless number and its value varies from 0 to 1 where zero signifies no change in stability whereas 1 indicates complete phase separation [55]. As shown in Figure 6, the instability index for all the emulsions increases with initial increase in rotational frequency (measured as rpm). The highest speed possible for the instrument was 4000 rpm, which is equivalent to 2300 g. Thus, in general, the emulsions prepared in this study are very stable even under high temperatures and rotational frequency. The instability index indicates that the emulsion in the salt solution is only affected by the applied conditions (e.g., rotational frequency and temperature), evident by the unchanging stability during time at any RPM. On the contrary, the emulsion in distillate water has an increasing instability index at high (>1000) RPM during the testing time at high (>1000) RPM. This trend confirms that the stability of the emulsions in the salt solution is better than in the distilled water. The temperature of the emulsions seems to have the more significant effect on stability in the salt solution, where the emulsion is clearly more unstable at high temperatures in the 1000 to 3000 RPM range. However, no clear trend can be drawn from the behavior observed over the range of the applied conditions. 

### 3.5. Stability of the Membrane Fibers

**Figure 7:**
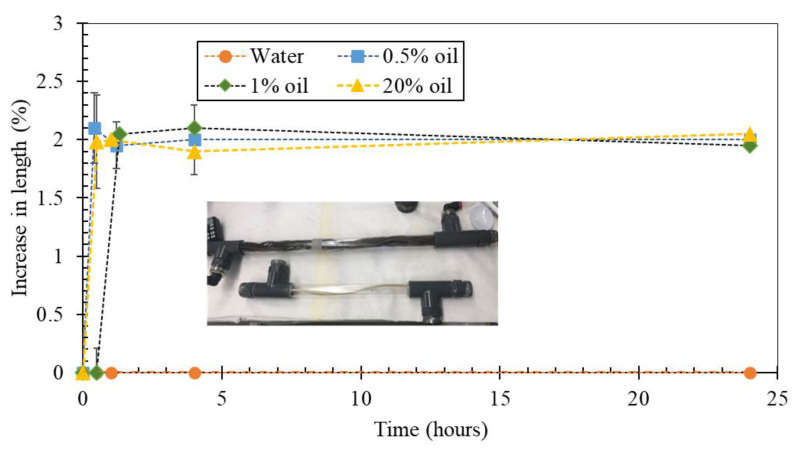
Increase in fiber length when exposed to the solutions containing different oil fractions (percentages).

It was observed that the shape of the fibers changed from straight to curly during ME experiments, as shown in the inset of Figure 7. In order to probe the reason behind the curling of the fibers, an experiment on the fiber stability when in contact with different solutions was carried out. The increase in the fiber’s length was measured over time when the fibers were immersed in different solutions (Figure 7). For all the solutions except distillate water, an increase in length of approximately 2% was observed and the fibers adopted a curly shape during the operation to accommodate the increased length. The increase in length occurred within the first hour or so and the fiber remained stable for the next 140 h. The fiber elongation (and, thus, curling) did not have any negative influence on the production of emulsions, but in future studies, the other membrane characteristics (pore size, mechanical strength) should also be analyzed after the experiments.

## 4. Implementation Perspectives 

Many of the chalk reservoirs found in the Danish part of the North Sea cannot handle PW directly as injection fluid for EOR. Hence, seawater has been considered as a smart injection fluid for potential EOR. However, if the PW can be treated to meet the requirements of the injection fluid, it can be a cheaper alternative to other injection fluids. Today, oil is removed from the water phase by separators, but our proposal is to use the PW with high oil content as the dispersed phase and transfer the oil into the PW with less oil content (continuous phase) via ME to produce tailored emulsions which then can be reinjected. 

The footprint and weight requirements of an ME plant were estimated based on the total amount (39,639 km^3^) of injection water needed in 2015 in the Danish part of the North Sea [31] and a total operation days of 350/year. Dan (injection water used 13,364 km^3^) and Halfdan (injection water used 10,760 km^3^)—two offshore rigs in the Danish part of the North Sea—were considered in order to estimate the requirements for each reservoir. Footprint and weight estimations were carried out according to the procedure described in the Appendix A. 

**Figure 8:**
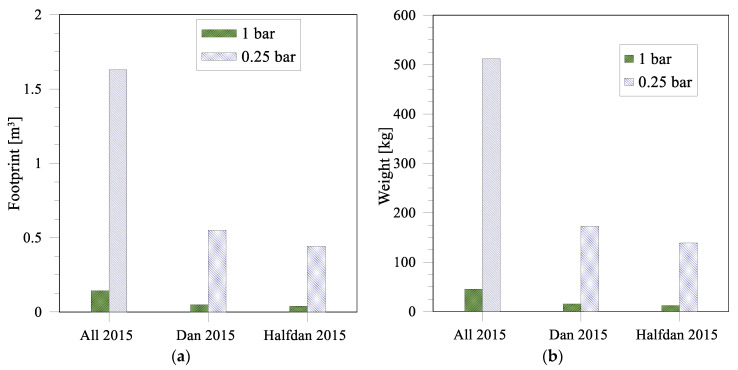
Membrane footprint (**a**) and weight (**b**) required to convert the daily volume of PW in the Danish part of the North Sea into emulsions for injection purpose.

The results show that the spatial footprint and weight required to convert the daily volume of PW used in Denmark into injection water vary from 0.04 to 1.6 m^3^ and 45–510 kg, respectively, depending upon the applied pressure (see Figure 8a,b, respectively)). If the injection water is divided into the different sites—here highlighted as the Dan and Halfdan sites—the space requirements for the membrane modules stay below 0.6 m^3^ in each case even for the lowest pressure considered in the current study. However, further optimization of the membrane flux and fouling analysis still needs to be carried out; thus, space requirements are expected to further decrease. 

The energy consumption is one of the main parameters that determines the operating cost of a process. Therefore, the analysis was extended to determine the specific energy consumption (SEC)—amount of energy required to produce one cubic meter of emulsion—of ME from the experimental data. The analysis considers only the energy required to circulate the pressurized (to-be-dispersed) phase, as the energy consumption associated with the other equipment (pump for the continues phase which operates at atmospheric pressure, magnetic stirrer, etc.) are relatively minor. SEC was calculated using the flow rate, applied pressure and the corresponding module productivity as follows [56]:

$$\mathrm{SEC}=\frac{Q\times \Delta P}{\left(J.A\right)36\times \eta }$$

where SEC is in kWh/m^3^, ΔP is the pressure applied on to-be-dispersed phase in bar, *Q* is the volumetric flow rate of to be dispersed phase and η is the efficiency of the pump (%/100).

SEC for ME working at 0.25, 0.5 and 1 bar are 3.1, 1.06 and 0.53 kwh/m^3^, respectively. It is interesting to note that the energy consumption decreases by increasing the applied pressure, which is a direct consequence of the large increase in flux with pressure (see Figure 2). SEC for the ultrasonic emulsification—a conventional emulsification technique—has been reported as high as 378 kWh/m^3^ [18], which demonstrates that the SEC for ME observed in the current study is more than 700 times less than the traditional emulsification techniques. 

## 5. Conclusions

Formation of crude-oil-in-water emulsions from produced water using ME was proposed as a novel alternative to its conventional treatments. The potential advantages of the proposed solution are twofold: elimination of the environmental impact associated with the discharge of produced water and potential utilization within the production process (e.g., enhanced oil recovery and demulsification of PW). It was found that the mean droplet size of the dispersed oil droplets could be tuned by varying the pressure on to-be-dispersed phase. Irrespective of the continuous phases (distilled water or 3.5% NaCl solution) used, the formed emulsions show very good stability even after days of storage and under different temperatures and pressures. The oil accumulated at the surface of the membrane was easily removable through alkaline cleaning; however, it was noted that the fibers became slightly curled after encountering the oily water, but the curling did not interfere with their performance. The energy of ME was more than almost three orders of magnitude less than the conventional ultrasonic emulsification. These observations, combined with footprint and weight analysis, show that ME presents a compact, lightweight and robust technology to produce crude-oil-in-water emulsions where the droplet size can be simply tuned by adjusting the operating pressure.

## Figures and Tables

**Table 1:** Description of the applied membrane and membrane module.

Membrane Material	Polypropylene
Type of membrane module	Hollow fiber
No. of fibers	19
Length of fibers (cm)	42
Inner fiber diameter (mm)	1.8
Outer fiber diameter (mm)	2.7
Membrane thickness (mm)	0.45
Average pore size (nm)	200
Porosity (%)	73
Surface area (cm^2^)	45

## Data Availability

The study did not report any data.

## Abbreviations

DI	(please define it in the manuscript) [appears first in Section 2.6]
DLS	Dynamic light scattering
EOR	Enhanced oil recovery
FR	(please define it in the manuscript) [appears in Figure 3 and Figure 4]
FTIR	Fourier Transform infrared
ME	Membrane emulsification
NaCl	Sodium chloride
PP	Polypropylene
PW	Produced water
RPM	Rotations per minute
SPG	Shirasu porous glass
TMP	Transmembrane pressure

## Appendix A

**Calculations of footprint and weight of ME unit**

The required membrane area, *A_m_*, is given by:

$$A_{m}=\frac{V_{iw}/2}{J}$$

where the volume of injection water (*V_iw_*) is divided by 2 since only the oil and water transport through the membrane fibers should be considered and not the continuous phase, which had an initial volume equal to the initial volume of the dispersed phase in this study, circulated on the other side of the membrane. The flux (*J*) obtained at 0.25 and 1 bar from the experiments (Figure 2) were used to calculate the membrane area.

The number of MD modules required, *N_m_*, and the total weight of modules, *W_tot_*, is given by:

$$N_{m}=\frac{A_{m}}{A_{1m}}$$

$$W_{t}=W_{1m}\cdot N_{m}$$

where *A*_1*m*_ and *W*_1*m*_ is the area and weight, respectively, of a single module.

The membrane module considered in this study was the DOWTM ultrafiltration module (Model SFP-2660) [57]. The weight of 1 module was 16 kg and the membrane module was 20.1 cm in diameter, 1 m in length and 34 m^2^ in area.

Finally, the size, S, of the system is:

$$S=N_{m}\cdot S_{m}\cdot \rho_{s}$$

where *S_m_* is the size of a single module and *ρ_s_* is the space density, which was considered to be 50%, and quantifies the packing of the modules into a container.
